# Can government-guided funds promote innovation output in strategic emerging enterprises? Evidence from China

**DOI:** 10.1371/journal.pone.0334826

**Published:** 2025-10-27

**Authors:** Chunyan Zhao, Lu Zhang

**Affiliations:** 1 School of Intellectual Property, Jiangsu University, Zhenjiang, Jiangsu, China; 2 School of Business, Nanjing University, Nanjing, Jiangsu, China; 3 School of Finance and Economics, Jiangsu University, Zhenjiang, Jiangsu, China; Southwestern University of Finance and Economics, CHINA

## Abstract

Using 9,138 observations from 731 strategic emerging enterprises in China’s A-share market between 2010 and 2023, this study explores the impact of government-guided funds on innovation in these enterprises and their underlying mechanisms. The main empirical findings are as follows: Firstly, there is a positive correlation between government-guided funds and innovation output in strategic emerging enterprises, and their endogeneity and robustness have been tested using instrumental variable methods and a series of other methods. Secondly, government-guided funds significantly enhance corporate innovation output through two pathways: one is by alleviating financing constraints on technological R&D; the other is by enhancing companies’ ability to access policy resources such as tax incentives. Thirdly, heterogeneity tests indicate that the innovative incentive effect of government-guided funds is more pronounced in eastern regions, state-owned enterprises, and companies with higher capital intensity from downstream customers. This study focuses on strategic emerging enterprises, providing a new perspective on the impact of government-guided funds on corporate innovation.

## Introduction

Against the backdrop of intensifying strategic competition between China and the United States, China’s industrial policy is facing growing international pressure: legal challenges within the World Trade Organisation (WTO) framework targeting market-distorting subsidy measures, and technological barriers such as semiconductor export controls [1]. These pressures are compelling China to establish a theoretically rigorous and internationally acceptable industrial policy framework to balance national development needs with the need to participate in global governance. China has positioned government-guided funds (GGF) as tools compliant with market rules, implementing industrial policy through decentralised, rule-based equity investments rather than direct subsidies, thereby advancing technological sovereignty while mitigating trade rule friction. The government-guided fund is the core practice of this new framework, which implements the planning goals of the country in strategic emerging industries through market-oriented capital operation and governance mechanisms. It is a key exploration to achieve the transformation of industrial policies from selective to functional. In this context, GGFs have become an important policy tool for stabilising the innovation ecosystem. GGFs are funds established by governments at all levels through budgetary arrangements. They can invest independently or in collaboration with social capital, using market-based methods such as equity investment to guide various types of social capital toward key areas and weak links in socio-economic development, thereby supporting the development of relevant industries and sectors. Their core feature lies in leveraging the multiplier effect and guiding role of fiscal funds. As of June 2024, according to the ‘China Government-Guided Fund Industry Market Analysis and Development Trend Forecast Report (2024),’ China has established 318 government-guided funds, managing total assets of RMB 4.52 trillion (approximately USD 623 billion) [2]. These funds aim to promote technological progress and industrial upgrading.

Existing academic researches have primarily focused on the economic performance evaluation of government venture capital (GVC) [3–5], but there has been a noticeable lack of discussion on the impact of China’s government-guided funds. It is important to note that while both GGFs and GVCs are supported by public finances, China’s GGFs feature a unique institutional design: local governments participate in market-oriented fund operations as limited partners, implementing industrial planning through governance coordination, thereby creating a Chinese-style policy tool that combines financial investment with strategic guidance. Different from the traditional government venture capital model popular in developed countries such as Europe and the United States, China’s GGF’s main goal is to solve market failure through temporary financial intervention, emphasizing financial sustainability and minimum distortion, reflecting unique institutional innovation, which is characterized by strategic embeddedness in industrial planning. While international GVCs typically operate at arm’s length from policy directives—either as passive limited partners in market-driven funds or as direct investors with strong independence—China’s GGFs integrate policy imperatives into their governance design. Local governments act as limited partners but retain substantial oversight through investment mandates, performance assessments linked to industrial objectives such as domestic re-investment ratios, and structured profit-concession mechanisms to incentivize private capital alignment with national priorities. This model transcends the traditional capital supply function of GVCs, emphasising the mitigation of innovation market failures through active governance, and highlighting stronger intentions for industrial upgrading and political attributes.

In recent years, a large number of literatures have studied the impact of government funds on enterprise innovation, but most of them are concentrated on all listed companies, specific industries (such as new energy) or regional innovation [6–8]. However, there is still no agreement on the actual impact and mechanism of government funds on innovation [9–12], and they fail to fully reveal the unique innovation mode and policy requirements of China’s strategic emerging industries. Strategic emerging industries play a vital role in supporting and promoting long-term economic development, and are key industries leading innovation driven growth [13]. According to the classification catalog of industrial strategic emerging industries (2023) issued by the National Bureau of statistics, strategic emerging industries mainly include nine areas: next generation information technology, advanced equipment manufacturing, new materials, biotechnology, new energy vehicles, new energy, environmental protection and energy conservation, digital creativity and related services. These industries have been designated as key cultivation fields by the state, representing the direction of future scientific and technological development, and playing a vital role in guiding and promoting the overall and long-term process of national economic and social development. Strategic emerging industries are facing unique challenges, especially in the entrepreneurial and early to medium-term stages. Companies often show high-risk characteristics, such as unclear technical approaches, low information transparency, and long investment return cycles. These characteristics lead to excessive concentration of venture capital in mature companies due to short-term profit seeking tendency, resulting in unbalanced allocation of market resources, and insufficient financing of early to medium-term innovation projects. Traditional policy tools such as financial subsidies may temporarily improve the capacity utilization of enterprises, but they may also lead to resource mismatch, adverse selection or rent-seeking behavior, prompting enterprises to put scale expansion above the improvement of innovation ability [14,15]. Therefore, the establishment of guidance funds through the market mechanism to attract social capital has become an important strategic choice to support the development of strategic emerging industries. When the technological complexity and return cycle exceed the spontaneous bearing capacity of the market, the government guided funds share the potential risks as public capital, so that the private sector can focus on R&D and rebuild the innovation incentive mechanism of strategic emerging enterprises through professional division of labor [16]. The traditional innovation theory based on general enterprise may not fully explain the innovation incentive in SEI [17,18], so it is necessary to explore the mechanism of GGF influencing the innovation achievements of strategic emerging enterprises.

This study utilises a dataset of strategic emerging enterprises listed on China’s A-share market from 2010 to 2023 to explore two core issues: the effectiveness of government-guided funds in promoting innovation among strategic emerging enterprises, and the impact of financing constraints, innovation uncertainty, information disclosure quality, and digital transformation on the promotional role of GGFs in driving innovation among these enterprises. Additionally, this paper analyses the heterogeneous effects of government-guided funds on innovation in strategic emerging enterprises across different strategic emerging industries and regions. The contributions of this paper are as follows: First, it enriches the existing research on the impact of government-guided funds on the innovative behaviour of micro-enterprises, particularly strategic emerging enterprises. This paper delves into the effects and mechanisms of government-guided funds on the innovation of strategic emerging enterprises, conducting a comprehensive and systematic examination of the dynamic influence of strategic emerging industry venture capital guidance funds on enterprise innovation from their initial regulatory phase to their current phase of vigorous development. This provides a clearer understanding of their effects and mechanisms on the innovation of micro-enterprises; Second, it validates the mechanism through which government-guided funds influence innovation in strategic emerging enterprises, further deepening our understanding of the nature and policy orientation of such funds. This has significant implications for clarifying the role of government-guided funds and leveraging their innovative leadership potential.

The structure of the following sections is as follows. Part 2 conducts theoretical analysis and proposes hypotheses. Part 3 describes the research design, including data sources and variable construction. Part 4 presents empirical results, including benchmark regression, moderating effects, robustness tests, and heterogeneity analysis. Part 5 summarises conclusions, proposes policy recommendations, and points out shortcomings.

## Theoretical analysis and hypotheses development

### The impact of government-guided fund on innovation

Government-guided fund is a key policy tool for addressing systemic market failures in the financing of strategic emerging industries (SEIs). This intervention logic is strongly supported by Aghion and Tirole’s public-private innovation model [16]. The model posits that outsourcing specialised R&D can resolve the inherent incentive mismatches in traditional financing structures, which primarily stem from the high uncertainty of technological innovation and severe information asymmetry. In this context, state capital is crucial for overcoming private investment shortfalls. Private capital tends to underinvest in SEIs because breakthrough technologies typically involve lengthy R&D cycles, high initial costs, and unpredictable commercial outcomes, thereby deterring firms from entering the market [17,19]. GGFs enhance firm innovation capabilities and bridge financing gaps through two channels: first, the government assumes residual risks while delegating operational autonomy to professional institutions. Government-guided funds enter enterprises through institutional investment, and the enterprises or projects they select for investment incorporate strategic directions from government decision-making, the market value of the enterprise, and expected future returns, thereby granting the invested enterprises and projects ‘government certification’ [20–22]. Investors typically possess strong signal-capturing capabilities, leveraging the professional sensitivity of government-guided funds to collect critical enterprise information by monitoring the positive industry signals they release, thereby alleviating information asymmetry between companies and external investors. Second, GGFs provide social resources for high-risk R&D [7,8,23–25]. Governments at all levels leverage fiscal budget funds through market-based equity investment mechanisms to channel social capital into strategic emerging industries, alleviating financing constraints during the innovation process and reducing the marginal cost burden of technological R&D. This, in turn, incentivises enterprises to expand the scale of their innovation investments and enhance output.

H1: Government-guided funds have a significant role in promoting innovation in strategic emerging enterprises.

### Mechanism effects

Technological innovation in strategic emerging industries has significant positive externalities, leading to insufficient spontaneous investment of social capital [26]. Government-guided funds leverage fiscal funds through market-oriented operations, effectively expanding the scale of risk capital supply [27], particularly addressing the financing constraints faced by early-stage enterprises. Their core role manifests in two aspects: first, the funds directly increase equity capital investment in innovative enterprises, alleviating financial pressures related to R&D equipment procurement and technical talent reserves; Second, they guide social capital toward high-risk original technology fields through policy orientation, correcting market imbalances where private capital is overly concentrated in mature enterprises. It is worth noting that some funds may reduce support for start-up enterprises due to risk-averse tendencies, potentially weakening their effectiveness in alleviating early-stage financing constraints [17]. However, overall, by expanding financing channels and reducing funding costs, this mechanism provides critical resource support for innovation output [28].

In addition, the investment behaviour of government-guided funds itself constitutes a market signal certification mechanism. The in-depth due diligence and investment decisions made by professional teams convey a credible signal of project quality to external investors [29]. This certification effect promotes innovation through two channels: first, it significantly reduces information asymmetry in the investment and financing market, enhancing the confidence of social capital to follow suit [30]. Invested companies are more likely to secure venture capital support in subsequent financing rounds, forming a sustainable financing chain that ensures the continuity of R&D investments. Second, it enhances the company’s ability to access policy resources. The implicit government-enterprise connection established through fund investment makes it easier for companies to obtain tax incentives and bank loans. Banks view government investment as a credit endorsement, reducing credit approval costs; simultaneously, companies can precisely access subsidies and other supportive measures through policy channels, indirectly alleviating the financial pressure of innovation activities.

Therefore, the following hypotheses are proposed:

H2a: Government-guided funds promote innovation and output among strategic emerging enterprises by alleviating financing constraints.

H2b: Government-guided funds promote innovation and output among strategic emerging enterprises by increasing the availability of tax incentives for enterprises.

## Data and methods

### Data

According to the National Bureau of Statistics’ ‘Catalogue of Strategic Emerging Industries in the Industrial Sector (2023),’ a list of strategic emerging enterprises was manually selected. The names of these enterprises were matched with investment event data from government-guided funds collected from the Zero2IPO Private Equity Pass database. Financial data for the remaining companies was sourced from the China Securities Market and Accounting Research (CSMAR) Database. The sample processing procedure is as follows: (1) Exclude samples with abnormal financial conditions, such as ST and *ST; (2) Exclude samples from the financial industry; (3) Perform linear interpolation on missing values; (4) Truncate all continuous variables at the 5% and 95% levels. A total of 9,138 observation data points were collected for 731 enterprises from 2010 to 2023. The sample in this study is limited to strategic emerging industries (SEIs) listed on the Chinese A-share market. Unlisted companies, especially early-stage SEI start-ups, typically face more severe financing constraints and information asymmetry issues. The government-guided fund support models they receive and the mechanisms through which GGFs influence their innovation may differ from those of listed companies. Due to data availability constraints, the conclusions of this study primarily apply to listed SEIs.

### Variables

The dependent variable innovation output (Innovation) is measured by taking the logarithm of the number of patent licenses granted to the company plus 1. The independent variable government-guided fund is set to 1 for the year in which the company first receives government-guided fund investment and subsequent years, and 0 for years prior to receiving investment. If the company does not receive government-guided fund investment, it is also set to 0. Following previous studies [30], control variables include Size, Lev, ROE, Intangible, Growth, and Mshare. Additionally, year and firm-level variables are controlled for. Use financing constraints and tax incentives as intermediary variables. The financing constraint variables are represented by the SA index, which is widely used in research on the Chinese capital market [6,31]. It only relies on company size and age, which are less affected by endogeneity issues compared to other financial ratio based indicators. This makes it suitable for the Chinese context as it avoids potential reverse causality where innovation output may simultaneously affect the company’s current financial ratios. In addition, its exogeneity relative to company performance makes it a target for capturing the external financing environment faced by strategic emerging enterprises in China. The variable of tax incentives is measured by the ratio of tax refunds received/ (tax refunds received + various taxes paid) [32], which is based on the company’s cash flow statement data and has good objectivity and comparability. It can effectively measure the actual cash tax refund intensity obtained by the company.The specific definitions of these variables are presented in Table 1.

**Table 1:** Variables definition and measurement.

Variable Type	Symbol	Measurement
Dependent variable	Innovation	The number of patents granted + 1 and then taking the logarithm
Independent variable	GGF	From 2010 to 2023, take 1 from the year when the enterprise first received government-guided fund support, and 0 for the rest
Control variables	Size	Natural logarithm of total assets for the year
	Level	Total liabilities at year-end / Total assets at year-end
	ROE	Net profit / Average balance of owners’ equity
	Intangible	Net intangible assets / Total assets
	Growth	Current year’s operating income / Previous year’s operating income - 1
	Mshare	Number of shares held by directors and supervisors / Number of total share capital
Mediating variables	SA	SA = - 0.737 Natural logarithm of firm’s total asset size + 0.043 (Natural logarithm of firm’s total asset size) (Natural logarithm of firm’s total asset size) - 0.040 Firm’s operating year
	Tax	Tax refunds received / (Tax refunds received + Various taxes paid)

### Model construction

This study uses Eq (1) to analyse the impact of government-guided funds on the innovative output of strategic emerging enterprises from 2010 to 2023.

$${\text{Innovation}}_{i,t}=\alpha +\beta {\text{GGF}}_{i,t}+\gamma X_{i,t}+\delta_{i}+\eta_{t}+\varepsilon_{i,t}$$ (1)

We formulate Eq (2) to examine how government-guided funds influence the innovation output of strategic emerging enterprises ([33]).

$${\text{y}}_{i,t}=\alpha +\beta {\text{GGF}}_{i,t}+\gamma X_{i,t}+\delta_{i}+\eta_{t}+\varepsilon_{i,t}$$ (2)

Where ${\text{y}}_{i,t}$ represents the mechanism variables, including financing constraints (SA) and tax incentives (Tax). In which subscripts *i* and *t* denote firm and year, respectively; Innovation signifies the firm’s invention; GGF indicates Government Guided Funds; X represents a collection of control variables. $\delta_{i}$ and $\eta_{t}$ denote the year and firm fixed effects, respectively. $\varepsilon_{i,t}$ represents the error term.

### Descriptive statistics

This study analysed relevant data from 731 strategic emerging enterprises between 2010 and 2023 to determine the unique characteristics of each variable, as shown in Table 2. The average innovation output of listed strategic emerging enterprises was 0.758, with a standard deviation of 1.581, indicating significant differences in technological innovation potential among listed strategic emerging enterprises. The standard deviation for enterprise scale is 1.281, indicating that strategic emerging enterprises exhibit significant differences in terms of scale, among other factors.

**Table 2:** Descriptive statistics.

Variables	Obs	Mean	SD	Min	Max
Innovation	9138	0.758	1.581	0.000	4.890
GGF	9138	0.067	0.251	0.000	1.000
Size	9138	22.330	1.280	19.078	27.245
Lev	9138	0.408	0.201	0.011	1.797
ROE	9138	0.048	0.640	-58.804	1.611
Intangible	9138	0.043	0.051	0.000	0.661
Growth	9138	0.207	1.120	-0.902	58.749
Mshare	9138	0.133	0.188	-0.045	3.002

## Empirical results

### Baseline results

Table 3 presents the research results. Model 1 does not include control variables, containing only firm-fixed effects and year-fixed effects. Model 2 reports the results with control variables included. The estimated coefficients of GGF are statistically significant at the 10% significance level and show an upward trend, confirming the effectiveness of government-guided funds in stimulating innovation output in strategic emerging enterprises. In summary, enterprises that received government-guided fund investments demonstrated a significant increase in innovation output compared to the control group.

**Table 3:** Baseline Results.

	Model 1	Model 2
GGF	0.246^*^	0.244^*^
	(1.91)	(1.86)
Control	NO	YES
Firm-FE	YES	YES
Year-FE	YES	YES
Observations	9138	9138
*R* ^2^	0.405	0.405

Note: The values within parentheses are standard errors clustered by firm; ^***^, ^**^, and ^*^ represent statistically significant at the level of 1%, 5%, and 10%.

### Mechanism effects

#### Alleviating financing constraints.

Model 1 in Table 4 reports the research results. The coefficient of GGF is positive and significant at the 1% significance level. This finding indicates that enterprises that obtain government-guided fund investment have more financial resources than those that do not receive such funding support. Therefore, they are able to overcome financing constraints and enhance innovation output, indicating that government-guided funds are crucial in promoting enterprise innovation. In summary, government-guided funds can effectively alleviate financing constraints and stimulate the input and output of enterprise technological innovation capabilities.

**Table 4:** Mechanism test results.

	Model 1	Model 2
	SA	Tax
GGF	0.029^***^	0.023^*^
	(2.71)	(1.75)
Control	YES	YES
Firm-FE	YES	YES
Year-FE	YES	YES
Cluster Firm	YES	YES
Observations	9138	9118
*R* ^2^	0.965	0.714

Note: The values within parentheses are standard errors clustered by firm; ^***^, ^**^, and ^*^ represent statistically significant at the level of 1%, 5%, and 10%.

#### Improve the level of tax incentives obtained.

The empirical results of Model 2 in Table 4 show that the coefficient of GGF for tax incentives is significantly positive at the 10% level. This result indicates that enterprises that receive investment from government-guided funds significantly improve their ability to obtain tax reduction and exemption policies through the qualification endorsement signal formed by government capital injections. The professional due diligence conducted by government-guided funds provides tax authorities with third-party certification of innovation authenticity, reducing information friction and administrative costs in tax incentive approvals. This enables enterprises to optimise the intertemporal allocation of R&D cash flows through tools such as tax credits and additional deductions. The certification effect of government-guided funds not only directly amplifies the leverage efficiency of fiscal funds but also addresses institutional failures caused by the positive externalities of innovation by strengthening the coupling of policy tools, ultimately achieving a systemic leap in the efficiency of technological innovation outcomes.

### Robustness test

#### Add city fixed effects.

To enhance the reliability of the research findings, city-level fixed effects were incorporated into the baseline regression model to control for unobserved heterogeneity at the city level, such as regional economic conditions, institutional environments, and policy differences. As shown in Model 1 and 2 of Table 5, the regression coefficients for GGF on innovation are all positive and significant at the 10% significance level. This finding indicates that even after controlling for city-specific factors, GGF still has a positive and economically significant impact on innovation in strategic emerging enterprises, thereby further consolidating the robustness of the benchmark results.

**Table 5:** Add city fixed effects.

	Model 1	Model 2
GGF	0.246^*^	0.244^*^
	(1.90)	(1.85)
Control	NO	YES
Firm-FE	YES	YES
Year-FE	YES	YES
City-FE	YES	YES
Cluster Firm	YES	YES
Observations	9138	9138
*R* ^2^	0.405	0.405

Note: The values within parentheses are standard errors clustered by firm; ^***^, ^**^, and ^*^ represent statistically significant at the level of 1%, 5%, and 10%.

#### IV-2SLS.

To address endogeneity issues, the average number of times other companies in the same industry received government-guided fund investments in the same year (IV) was used as an instrumental variable [34]. Table 6 presents the regression results of the endogeneity test. The results indicate that the coefficient of the independent variable ‘GGFs’ consistently exhibits positive significance at the 10% level, suggesting that the establishment of GGFs significantly promotes innovation output in strategic emerging enterprises, consistent with the baseline regression results. Additionally, the corresponding p-value of the Kleibergen-Paap rk LM statistic is significantly below the 1% significance level, passing the unidentifiability test. The Cragg-Donald Wald F statistic is 48.74, significantly exceeding 10, indicating no weak instrumental variable issue.

**Table 6:** IV-2SLS test.

	Model 1	Model 2
	The first stage	The second stage
IV	0.199^**^	
	(2.15)	
GGF		3.565^*^
		(2.35)
Control	YES	YES
Firm-FE	YES	YES
Year-FE	YES	YES
Cluster Firm	YES	YES
Observations	9138	9138
*R* ^2^	0.609	-0.180

Note: The values within parentheses are standard errors clustered by firm; ^***^, ^**^, and ^*^ represent statistically significant at the level of 1%, 5%, and 10%.

#### Parallel trend test.

Using event analysis, we explore the dynamic impact of government-led funds on the innovative output of strategic emerging enterprises [35]. It should be noted that there are few observations for the five years or more before and after a firm receives investment from a government-guided fund, which may lead to biased estimates. Therefore, we merged these observations for analysis and using the situation in the year prior to the investment as the comparison benchmark, thus omitting the case where j = -1 [36]. Eq 3 builds upon Eq 1 by introducing an interaction term between policy dummy variables and time dummy variables to analyse the impact of government-guided fund investments during a specific period before and after the investment.

$${\text{Innovation}}_{i,t}=\alpha +\beta \sum_{j\neq -1}{\text{GGF}}_{i,t-j}+\gamma_{j}X_{i,t}+\delta_{i}+\eta_{t}+\varepsilon_{i,t}$$ (3)

**Fig 1:**
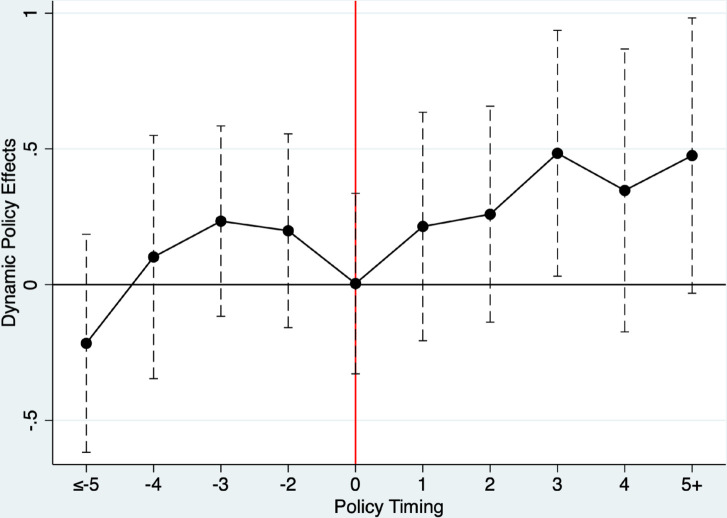
Parallel trend tests.

Where the subscript *j* denotes the relative year in which the merger or acquisition was completed. For example, j≠−1 indicates the year prior to the completion of the merger or acquisition. Therefore, ${\text{GGF}}_{i,t-j}$ takes a value of 1 when company *i* receives investment from the government-guided fund in the relative *jth* year of year *t*, and 0 otherwise. This approach essentially decomposes the average treatment effect across different years to better observe the specific performance of the estimated results before and after the investment. Fig 1 presents the research findings. The study reveals that the current impact of government-guided fund investments has weakened compared to previous periods, primarily due to the lag effect of such investments. Following government-guided fund investments in enterprises, these enterprises’ financial investments in innovation and R&D continued to grow. Therefore, the data samples rigorously tested and confirmed as reliable in this study successfully passed the parallel trend test, thereby enhancing the reliability of the empirical model and its corresponding results. This indicates that the allocation of government-guided funds has a significant positive impact on enhancing enterprise innovation output.

### Heterogeneity analysis

#### Diverse geographical distribution of enterprises.

Models 1 and 2 in Table 7 examine whether the impact of government-guided funds on the innovative output of strategic emerging enterprises varies by geographical location. The analysis focuses on the heterogeneity of the impact of technological innovation among the sample enterprises, which are divided into two regions: eastern and central-western [37]. The research findings indicate that government-guided funds have heterogeneous effects on the innovative outcomes of enterprises in different geographical regions. The coefficient of GGF is significantly positive in the eastern region, indicating that government-guided funds have the most significant incentive effect on the innovative output of enterprises in the eastern region. Additionally, the coefficient of GGF is not significant in the central and western regions, indicating that government-guided funds do not have a noticeable effect on the innovative output of enterprises in these regions. This may stem from differences in regional institutional environments and the completeness of innovation factors between the eastern and central/western regions. The dense concentration of universities and supply chain support in eastern regions enables policy funds to be efficiently converted into R&D outcomes; however, in central and western regions, the lack of technology transfer channels, incomplete industrial chains, and weak market mechanisms result in policy resources failing to effectively stimulate enterprise innovation momentum.

**Table 7:** Heterogeneity test results.

	Model 1	Model 2	Model 3	Model 4	Model 5	Model 6
	Region		Ownership		SellDemand	
	Eastern regions	Central and Western regions	Non-state-owned firms	State-owned firms	Low-level	High-level
GGF	0.348^**^	0.005	0.157	0.385^*^	0.227	0.371^*^
	(2.09)	(0.03)	(0.91)	(1.86)	(1.21)	(1.96)
Control	YES	YES	YES	YES	YES	YES
Firm-FE	YES	YES	YES	YES	YES	YES
Year-FE	YES	YES	YES	YES	YES	YES
Cluster Firm	YES	YES	YES	YES	YES	YES
Observations	6764	2373	6069	3055	4499	4540
*R* ^2^	0.402	0.435	0.406	0.435	0.437	0.460

Note: The values within parentheses are standard errors clustered by firm; ^***^, ^**^, and ^*^ represent statistically significant at the level of 1%, 5%, and 10%.

#### Different ownership.

The sample companies are classified into non-state-owned enterprises and state-owned enterprises based on ownership, as shown in Models 3 and 4 of Table 7. The research results indicate that government-guided funds have a positive impact on innovation investment for both state-owned enterprises and private enterprises. Government-guided funds have a substantial impact on the innovation output of state-owned enterprises, with a coefficient of 0.383 for GGF, which is significant at the 10% significance level. However, the impact on non-state-owned enterprises is not significant. This may be because SOEs, after receiving government-guided funds, have greater capacity to attract resources that promote innovation and can effectively incentivise innovation output. On the other hand, non-state-owned enterprises have relatively limited capacity to attract high-quality innovation resources, leading to an inability to successfully carry out specific innovation projects within the company, thereby resulting in lower innovation output performance compared to SOEs.

#### Different levels of capital utilisation among downstream customers.

To examine whether the impact of government-guided funds on innovation outcomes is influenced by the degree of cash flow constraints on enterprises, this paper conducts a grouped test based on the level of customer fund occupation. The ratio of total accounts receivable, notes receivable, and prepayments to main business revenue (SellDemand) is used as an indicator to measure the extent to which downstream customers in the supply chain occupy the company’s funds ([38]. The smaller the value of this indicator, the less customer arrears there are, which helps maintain normal cash flow and provides the necessary financial support for the company’s technological innovation.

As shown in Models 5 and 6 of Table 7, the sample is divided into low and high customer funding occupancy groups based on the median value of SellDemand. The results show that government-guided funds have a significant incentive effect on corporate innovation output in the high capital occupation level group, but the incentive effect is not significant in the low capital occupation level group. This may be because the innovation activities of strategic emerging enterprises are highly capital-dependent and high-risk, and the depth of customer capital occupation shapes the differences in their internal financing capabilities. High capital utilisation enterprises are caught in a double bind of cash flow shortages and external financing exclusion, facing hard budget constraints on innovation investment. At this point, the intervention of government-guided funds directly breaks the innovation bottleneck by supplementing scarce capital and improving credit signals. In contrast, low capital utilisation enterprises have robust cash flow cycles that already support routine R&D investment, and the marginal increment of policy funds is unlikely to trigger additional innovation responses. Additionally, the promotional effect of government capital on innovation outcomes is also a process of rebalancing the power structure within the supply chain. Customer capital occupation essentially represents the downstream’s ability to extract value from core enterprises through bargaining power. The resulting cash flow disruptions propagate innovation-inhibiting effects along the supply chain. Companies in the high capital occupation group, due to delayed payments from downstream entities, are forced to reduce investments in upstream technical cooperation and R&D collaboration, forming a supply chain innovation bottleneck; In this context, government-guided funds can reconstruct the efficiency of upstream-downstream capital flow coordination through targeted capital injections, effectively resolving operational capital bottlenecks at node enterprises and enabling companies to resume resource investments in innovation networks. Conversely, companies in the low capital occupation group have already maintained a stable state of innovation collaboration through healthy supply chain credit cycles, making policy intervention marginally effective and resulting in limited incentive effects.

## Conclusions

Based on a comprehensive analysis of 9,138 A-share listed strategic emerging enterprises in China from 2010 to 2023, this study confirms that government-guided fund is a key policy tool for addressing systemic market failures in strategic emerging industries, significantly enhancing corporate innovation output. Empirical results indicate that GGFs significantly promote innovation activities among SEIs, confirming their effectiveness in overcoming the dual challenges of high innovation uncertainty and severe information asymmetry inherent in SEIs. This paper validates this conclusion through a series of robustness tests. GGFs mitigate financing constraints, enhance companies’ ability to access tax incentive policies, and incentivise innovation output in strategic emerging enterprises. The promotional effects of GGFs on innovation in strategic emerging enterprises exhibit heterogeneous impacts across different regions, property rights structures, and the intensity of downstream customer capital occupation.

This paper proposes three policy recommendations. First, the leverage function of government-guided funds in regional coordination should be strengthened, with risk compensation mechanisms and talent recruitment policies tailored to address the innovation shortcomings of enterprises in central and western regions; Second, the governance structure of funds should be optimised by setting differentiated performance metrics to reduce the pursuit of short-term returns, particularly by increasing tolerance for extended investment cycles for enterprises with high capital intensity of downstream customers, thereby ensuring the sustainability of original technology R&D. Additionally, a dedicated empowerment channel for private enterprises should be established, combining tax incentive conversion service platforms to break down resource access barriers, ensuring that policy benefits precisely reach innovation entities, and fully unleashing the innovation potential of strategic emerging industries.

This study acknowledges two main limitations. First, the research focuses solely on A-share listed companies, neglecting unlisted SEI startups, which face more severe financing gaps than listed companies, potentially leading to an underestimation of GGF’s ability to correct market failures. Additionally, China’s unique institutional analysis cannot assess whether the observed political capital substitution mechanism is comparable under other governance frameworks, such as the U.S. CHIPS Act or the EU’s Green Industry Plan.
